# Caregiving Artificial Intelligence Chatbot for Older Adults and Their Preferences, Well-Being, and Social Connectivity: Mixed-Method Study

**DOI:** 10.2196/65776

**Published:** 2025-03-13

**Authors:** Brooke H Wolfe, Yoo Jung Oh, Hyesun Choung, Xiaoran Cui, Joshua Weinzapfel, R Amanda Cooper, Hae-Na Lee, Rebecca Lehto

**Affiliations:** 1 Department of Communication Michigan State University East Lansing, MI United States; 2 Brian Lamb School of Communication Purdue University West Lafayette, IN United States; 3 Department of Communication University of Connecticut Storrs, CT United States; 4 College of Engineering Michigan State University East Lansing, MI United States; 5 College of Nursing Michigan State University East Lansing, MI United States

**Keywords:** older adults, technology use, AI chatbots, artificial intelligence, well-being, social connectedness, mobile phone

## Abstract

**Background:**

The increasing number of older adults who are living alone poses challenges for maintaining their well-being, as they often need support with daily tasks, health care services, and social connections. However, advancements in artificial intelligence (AI) technologies have revolutionized health care and caregiving through their capacity to monitor health, provide medication and appointment reminders, and provide companionship to older adults. Nevertheless, the adaptability of these technologies for older adults is stymied by usability issues. This study explores how older adults use and adapt to AI technologies, highlighting both the persistent barriers and opportunities for potential enhancements.

**Objective:**

This study aimed to provide deeper insights into older adults’ engagement with technology and AI. The technologies currently used, potential technologies desired for daily life integration, personal technology concerns faced, and overall attitudes toward technology and AI are explored.

**Methods:**

Using mixed methods, participants (N=28) completed both a semistructured interview and surveys consisting of health and well-being measures. Participants then participated in a research team–facilitated interaction with an AI chatbot, Amazon Alexa. Interview transcripts were analyzed using thematic analysis, and surveys were evaluated using descriptive statistics.

**Results:**

Participants’ average age was 71 years (ranged from 65 years to 84 years). Most participants were familiar with technology use, especially using smartphones (26/28, 93%) and desktops and laptops (21/28, 75%). Participants rated appointment reminders (25/28, 89%), emergency assistance (22/28, 79%), and health monitoring (21/28, 75%). Participants rated appointment reminders (25/28, 89.3%), emergency assistance (22/28, 78.6%), and health monitoring (21/28, 75%) as the most desirable features of AI chatbots for adoption. Digital devices were commonly used for entertainment, health management, professional productivity, and social connectivity. Participants were most interested in integrating technology into their personal lives for scheduling reminders, chore assistance, and providing care to others. Challenges in using new technology included a commitment to learning new technologies, concerns about lack of privacy, and worries about future technology dependence. Overall, older adults’ attitudes coalesced into 3 orientations, which we label as technology adapters, technologically wary, and technology resisters. These results illustrate that not all older adults were resistant to technology and AI. Instead, older adults are aligned with categories on a spectrum between willing, hesitant but willing, and unwilling to use technology and AI. Researchers can use these findings by asking older adults about their orientation toward technology to facilitate the integration of new technologies with each person’s comfortability and preferences.

**Conclusions:**

To ensure that AI technologies effectively support older adults, it is essential to foster an ongoing dialogue among developers, older adults, families, and their caregivers, focusing on inclusive designs to meet older adults’ needs.

## Introduction

### Background

Both global life expectancy and the aging population have continued to increase in recent years [1]. As of 2023, 1 billion people in the world were aged 60 years or older and numbers are expected to rise to 1.4 billion by 2030 [2]. This trend presents challenges for maintaining the well-being of older adults, many of whom choose independent living while requiring assistance with daily tasks and health care routines, as well as support to stay socially connected [2,3].

Advancements in artificial intelligence (AI) technologies have transformed numerous sectors, including health care and caregiving (eg, supporting people with dementia) [4]. AI tools are now being used to monitor health, provide reminders for medication and appointments, and even offer companionship through conversational agents [5,6].

Despite the growing interest and potential in leveraging AI for older adult populations, challenges remain, particularly regarding the suitability of these technologies for older adults. One major issue is the limited accommodation for the unique needs of older adults, such as user-friendliness for those with limited experience using digital resources [7]. Security and privacy concerns are also significant barriers that can hinder older adults’ willingness to adopt AI agents for personal health care [8]. Finally, limited research on their long-term effectiveness underscores the need for more studies to ensure these technologies are sustainably introduced and adopted by older people [4,5].

Acknowledging the need for a personalized, older adult-friendly system that addresses the challenges and needs of older adults in their daily lives, this study aims to explore older adults’ current use, challenges, and desired needs associated with AI technology, specifically focusing on caregiving chatbots. As a result, this study seeks to provide a groundwork that will inform the development of AI technologies tailored to improve the well-being of older adults.

### AI Technologies in Health Care and Caregiving for Older Adults

While older adults use technology less frequently and in fewer ways than younger individuals, they are still capable of engaging with specific technologies that meet their needs [9,10]. Voice-based chatbots, for example, have been shown to help with medication adherence and reducing loneliness and social isolation, provided they are designed with older users in mind [8,11,12].

The rapid advancement of multimodal large language models like ChatGPT (OpenAI) suggests that many existing barriers for conversational agents in health and well-being may soon be overcome [13,14]. These advancements are expected to enhance conversational competence, domain-specific information, and personalization. Such progress can potentially address older adults’ concerns with existing technologies and improve their experience using these technologies.

Recent developments have focused on creating AI technologies specifically for older adults, aiming to address their needs for companionship, support, and health management. SeniorTalk [15], for example, offers an AI-powered chat companion that can be personalized through the selection of different personas. ElliQ (Intuition Robotics) is a social robot that uses conversational AI to engage older adults in daily activities, provide reminders, and facilitate social interaction to help reduce loneliness [16]. These technologies reflect the ongoing efforts to adapt AI applications to the specific needs of older adults. However, the deployment of AI in social care must be approached cautiously, ensuring that these technologies are implemented in ways that uphold the rights and dignity of older individuals [17].

While previous studies have explored older adults’ needs and concerns regarding technology use and the requirements for technology to assist aging through input from stakeholders like care professionals, technology designers, and policymakers, these insights have not been fully reflected in the development of technology [18,19]. Additional insight into older adults’ preferences for using technology is needed to tailor technological tools to benefit older users.

### Social Support and Older Adults’ Health and Well-Being

AI technology carries the potential as a source of social support that is greatly needed among the most vulnerable older adult population. Approximately a quarter of older adults live in social isolation and approximately one-third of older adults experience loneliness [20,21]. Loneliness is associated with many negative consequences including poor mental and physical health, lower self-rated well-being, and disrupted sleep quality [19]. Among older adults specifically, loneliness is also associated with reduced physical activity, impaired cognition, dementia progression, nursing home placement, and higher mortality rates [22]. Older adults with limited social networks and low social engagement are at greatest risk [23]. AI chatbots may provide a valuable source of companionship and social support, potentially addressing loneliness. The current study explores the technologies older adults currently use, what they are interested in integrating into their daily lives, the challenges they face, and their overall attitudes toward technology and AI. To examine these topics, 4 research questions are addressed:

RQ1: In what ways do older adults report using technology and AI?

RQ2: What technology and AI are older adults interested in integrating into their daily lives?

RQ3: What concerns and challenges do older adults report about technology and AI use?

RQ4: What are older adults’ attitudes toward technology and AI?

## Methods

### Participant Recruitment and Data Collection

A mixed methods cross-sectional descriptive design was used. Participants were recruited using the website of a community research pool, a research participation system, managed by a large midwestern university. Participants needed to meet these criteria: (1) at least 65 years old and (2) able to complete the interview in person at the university site. The community research pool system posted a short synopsis of the study on their website including the inclusion criteria, compensation for participants for completing the study qualification survey and a 45-minute interview, and researcher contact information. Participants selected an interview date and time through the community research pool system. Once a time was selected, a member of the research team reached out to confirm the interview schedule, provide instructions for the interview location (conference room at a university) and parking, and answer any questions through email. A total of 33 participants signed up for the study, with 5 dropping out before the interview due to scheduling conflicts, resulting in 28 participants who participated and completed the study.

After arriving for their interview, participants completed informed consent followed by a survey that included demographics, health, and well-being measures on either an iPad (Apple Inc) or paper, depending on personal preference. The participants then completed the interview with 2 members of the research team. One member led the interview by asking protocol questions. The interview was divided into questions about daily schedules (eg, What are routines that you do every day?), knowledge of technology and AI (eg, What technology do you use daily?), and social support desired for technology and AI (eg, What, if any, kind of challenges do you face when learning new technologies?). The second team member facilitated the participant’s interaction with an AI chatbot, Amazon Alexa. The participant was prompted to acknowledge the chatbot and then use multiple features, including asking for a recipe, playing a trivia game, and requesting the news from Alexa. Pseudonyms were assigned to each participant following the interview. The research team consisted of 5 women faculty members (PhD), 1 woman who is currently a graduate student, and 1 man who is currently an undergraduate student. The lead researcher had previously conducted multiple qualitative studies involving interviews. No previous relationship was established with participants before the interview. Researchers informed participants that the study was a part of a research project and disclosed their professional interests. Audio recordings were collected and transcribed for data analysis. They were not returned to participants for comments.

### Statistical Analysis

Descriptive statistics were used for analyzing the surveys. A thematic analysis of the interview data was conducted following phases of reflexive thematic coding, which included data familiarization, systematic coding, generating initial themes, developing and reviewing themes, refining and naming themes, and writing the manuscript [24]. To ensure the robustness of our analysis, we used 5 verification procedures: referential adequacy, peer debriefing, negative case analysis, audit trail, and exemplar identification [25,26]. Initially, we split the data to achieve referential adequacy and, after reaching saturation in the first half, we analyzed the second half of the data and did not find any new themes [27]. The first and fourth authors held 2 peer debriefing meetings to resolve differences and reach consensus on all themes. Continuous peer debriefing occurred throughout the analysis, and labels for the findings were determined collaboratively. We refined our analysis to account for all data, meeting the standard for negative case analysis [25]. Detailed notes were kept (ie, an audit trail), informing both the analysis and the selection of exemplars [26].

### Survey Measures

The study collected data on the following measures: demographics, general health status, Behavioral Risk Factor Surveillance System (BRFSS) Disability Questions, loneliness (3-item Loneliness Scale), life satisfaction (The Satisfaction with Life Scale) listed in Table 1 and digital literacy (Digital Health Technology Literacy Assessment Questionnaire), AI literacy, and interests in AI chatbot features for caregiving assistance, reported in Table 2 [28-32].

**Table 1:** Quantitative summary of older adults’ health and assistive technology use reported in study survey (N=28).

Health factors	Values
Health status, mean (SD)	3.6 (0.9)
Limited in activities, n (%)	9 (32)
Use Alexa as an assistive technology, mean (SD)	3.6 (1)
Loneliness, mean (SD; range)	4.1 (1.6; 3-8)
Life satisfaction, mean (SD; range)	3.9 (0.7; 2-5)

**Table 2:** Quantitative summary of older adults' current technology use reported in the study survey (N=28).

Item		Responses, n (%)
AI^a^ chatbot use		
	Amazon Alexa	10 (36)
	Google Assistant	6 (21)
	Apple Siri	9 (32)
	Facebook Messenger Bot	3 (11)
	ChatGPT	2 (7)
	Copilot	1 (4)
Challenges using technology devices		
	Never	2 (7)
	Rarely	6 (21)
	Sometimes	16 (57)
	Often	3 (11)
	Always	1 (4)
Knowledge about AI		
	Don’t have much knowledge	9 (32)
	Have knowledge	19 (68)
AI chatbots’ caregiving features interested in adopting		
	Appointment reminder	25 (89)
	Information access (eg, news, weather)	22 (79)
	Emergency assistance	22 (79)
	Health monitoring	21 (75)
	Daily medication reminders	21 (75)
	Fall detection and alert notifications	18 (64)
	Interactive entertainment	17 (61)
	Social connection	17 (61)
	Shopping management	17 (61)
	Physical activity encouragement	13 (46)
	Home automation control	13 (46)
	Mental health support	11 (39)
	Customized health tips and reminders	11 (39)
	Daily routine assistance	11 (39)
	Memory aids	11 (39)
	Dietary assistance	9 (32)
	Language and communication	2 (7)

^a^AI: artificial intelligence.

### Ethical Considerations

The institutional review board at Michigan State University determined that the study was exempt before study recruitment was initiated (STUDY00010214). Each person consented to participate in the study at the beginning of the survey and again at the beginning of their interview. Participants were informed that they could end the interview at any time and could skip any questions they did not feel comfortable answering. Each interview was recorded through Zoom (Zoom Communications Inc). The transcripts were reviewed, and all identifiable information was removed during the transcription process to protect participant information. Participants chose between US $50 cash or an Amazon gift card of the same amount as compensation for their time.

## Results

### Survey Findings

Among 28 participants, the average age was 71 years (SD 5.5), ranging from 65 to 84. More than half identified themselves as women (57%,16/28) and married or living with partners (61%, 17/28). The remaining participants were widowed, divorced, or separated (29%, 8/28) or never married or single (11%, 3/28). Most participants were living with their partner (61%, 17/28) or their partner and adult children 7% (2/28). Some participants reported that they were living alone (29%, 8/28). Almost all participants were white (89%, 25/28), followed by Asian (7%, 2/28), and black orAfrican American (4%, 1/28). Most participants were currently unemployed or retired (75%, 21/28). The highest level of education obtained by participants was, 43% (12/28) had completed high school, general educational development, or some college, while 57% (18/28) were college graduates or had attained a higher level of education. Participants’ household income levels were under US $50,000 (25%, 7/28), between US $50,000 and US $100,000 (36%, 10/28), between US $100,000 and US $150,000 (29%, 8/28), and above US $150,000 (4%, 1/28).

Tables 1 and 2 present a detailed report of participant profiles, including older adults’ health factors, technology and AI use, challenges, knowledge, and their intention to adopt AI features to support daily health routines. Participants’ average health status was 4 (SD 0.9) which was deemed to be good (from 1 being poor to 5 being excellent), and 32% (9/28) reported that they are limited in activities due to physical conditions. The average loneliness felt by the participants was 4 (SD 1.6) [Range 3-8] which indicated that participants felt lonely some of the time. Life satisfaction was 4 (SD 0.7) [Range 2-5] meaning that participants generally were satisfied with their lives.

Most participants were generally familiar with using technology. For chatbot use, 64% (18/28) reported that they have previously used AI chatbots. Participants were generally comfortable with interacting with a chatbot (mean 4, [SD 1.0], range 2-5). Regular use of technology devices was mostly related to smartphone (93%, 26/28) and desktop use (75%, 21/28), followed by iPad or tablet (46%, 13/28). Other types of technology included smartwatch (14%, 4/28), robot vacuum cleaner (18%, 5/28), and robot lawnmower (4%, 1/28). For regular application use, 75% (21/28) used social media, 71% (20/28) used online shopping, 64% (18/28) used banking and streaming services, 57% (16/28) health application, and 39% (11/28) used customer service chatbots. Participants reported that they face challenges using technology devices never (7%, 2/28), rarely (21%, 6/28), sometimes (57%, 16/28), often (11%, 3/28), and always (4%, 1/28). 32% (9/28) reported that they do not have much knowledge about AI and 68% (19/28) reported to have knowledge about AI.

When participants were asked if there were any AI chatbots’ caregiving features they may be interested in adopting, appointment reminder was rated the highest (89%, 25/28), followed by information access such as news or weather (79%, 22/28), emergency assistance (79%, 22/28), health monitoring (75%, 21/28), daily medication reminders (75%, 21/28), fall detection and alert notifications (64%, 18/28), interactive entertainment (61%, 17/28), social connection (61%, 17/28), shopping management (61%, 17/28), physical activity encouragement (46%, 13/28), home automation control (46%, 13/28), mental health support (39%, 11/28), customized health tips and reminders (39%, 11/28), daily routine assistance (39%, 11/28),  memory aids (39%, 11/28), dietary assistance (32%, 9/28), and language and communication (7%, 2/28).

### Qualitative Findings

#### Overview

Older adults discussed their current and desired use of technology and AI in their daily lives. These discussions were depicted alongside key concerns and challenges that impacted the degree to which participants were willing to accept and integrate these technologies. These considerations informed the construction of 3 distinct attitudes toward technology and AI. These findings respond to 4 research questions outlined.

#### RQ1: Current Technology and AI Use

The most common technologies participants mentioned are digital devices such as smartphones, smartwatches, computers, and radios (Table 2). These technologies were used for a range of purposes, including entertainment, health management, professional productivity, and social connectivity, illustrating that older adults used these technologies to enhance their quality of life, manage health, and mitigate social isolation.

Many participants reported using a combination of digital devices smartphones, smartwatches, and computers that are often integrated. For instance, Everett mentioned, “I have an iPhone, an iMac, and an iPad.” These interconnected ecosystems facilitated a convenient and useable community of digital devices that allowed older adults to engage online. In addition, older adults commonly use radio applications and devices to listen to stories, news, and music. Dennis expressed, “You know, I hear all these things on the radio. I listen to the radio every day.” The digital devices used by older adults facilitated their existing implementation of these technologies for social interaction, entertainment, health management, and aiding productivity. Each of these applications is overviewed in Table 2.

#### RQ2: Interested Integration

Older adults also reflected on areas of their lives where they were not currently using technology but would consider the addition of technology as helpful. Participants communicated that technology would be beneficial for scheduling reminders, chore assistance, and providing care to others. These categories illustrate how older adults desire to use technology to maintain their independence and manage daily tasks more effectively. These findings offer insights into the type of tasks proposed as helpful, outlined in Tables 3 and 4.

**Table 3:** Examples of current technology use that older adults described in the study interview.

Current technology use		Examples
**Social interaction**		
	Technology helps older adults maintain social connections through social media, email, and video calls, combating feelings of isolation.	“I guess, I’m in a LGBT and also friendship group…. it’s a group of gay people, primarily men who meet once a month at a different restaurant, just a friendship group to explore a different restaurant[s]...very excited.” (Raymond) “it’s just kind of our normal. Sunday, you know, church stream. I would say I went more in person before the pandemic, more so before the pandemic. Yeah, and we’ve all changed our habits I think since then.” (Christine)
**Entertainment**		
	Older adults engage with technology content, like streaming movies, listening to music, and playing games to stay mentally active.	“...it’s radio. Sometimes you can, it just gives out the full like the visual kind of like a video version of the news as well. So, it just depends on what you want, you know, like, you can ask ABC news.” (Cheryl) “Well, most of my downtime I probably spend on YouTube because there’s terrific history and documentary channels that I follow….I [also] read all my news and everything on online with. I go to a bunch of the different websites so I can get a wider perspective on what people are thinking.” (Ingrid)
**Health management**		
	Technologies can assist older adults in health-relates tasks, such as scheduling doctor’s appointments, tracking fitness, and managing medication efficiently.	“I wear hearing aids, and they’ve got Bluetooth on them, so, you know, I can listen to music through my phone and stuff like that.” (Sandra) “I set my phone as soon as I’ve eaten, and I reset it for two hours intervals. And then it has a reminder on it to take my vitamins.” (Gwen)
**Assisted productivity**		
	Older adults use devices professional activities like remote work, scheduling, and task management.	She’s [Alexa] a good reminder-er. I mean, I’ve got to tell her to, feel free to tell me to hurry up and sent out Easter cards, because Easter cards really soon….you know can I put egg shells down the garbage disposal and she’s No no no no no no, don’t do that. I didn’t really remember that I couldn’t do that. (Devorah) “So, there’s a lot of communication on my phone to them [church group]. I’m in a group of about 500 people, and there’s usually things going on with them. Like, right now, we have a friend who’s in the hospital dying, so we’re making arrangements for him. Just a lot of arranging. You know, I’m a connector person….I’m the head of a couple groups, so sometimes people contact me through email.” (Donna)

**Table 4:** Example of desired technology use that older adults described in the study interview.

Desired technology use			Example
**Scheduling reminders**			
	Older adults want some tools to help them stay organized by reminding them of tasks and events, thus supporting their daily routines and commitments	[“I use] auto bill pay I never go to the bank. Everything is auto paid. It’s, I mean prescriptions. I only take one, but, you know, it just pops into your thing [phone].” (Mary) “….at work and I do have a medication [reminder], and some reminders on my phone that remind me to turn in my time sheet or a medication reminder. Yeah, reminders that I need to take my medication, a second medication.” (Joyce)	
**Chore assistance**			
	Older adults are interested in technologies that simplify and automated daily chores to enhances convenience and reduce effort, such as vacuuming, mowing driving cleaning, and cooking.	“I would love to not have to mow my lawn. If that was automated, that would be awesome. See, I would love not to have to cook. That would be great. Basically, I’d like to live in the jetsons…it would be great to have somebody tale your dishes and not have to do that.” (Ingrid) “So, you know, if something would just automatically cook my food for me, just what I want for dinner without it being Grubhub or Uber Eats or somethings. Yeah, just, you know, my own food because I like my own cooking.” (Christine)	
**Caregiving monitoring**			
	Older adults expressed interest in technology/AI that would assist them in observing, supervising, and managing the care of others in their life. Participants clarified that they might want caregiving assistance in the future, but at this point, they were focused on completing care tasks for others including their own parents or partner.	“More support? Not yet…I mean, I drove here…[but] a device for who could tell if I if I had fallen down without me having to hit a button or anything…but that that’s something we would. Yeah. I’d be interested in.” (Timothy) “It has to do with my caregiving…I’m always trying to find things that might help. My mom and I found online I heard a story on the radio about this company called LEC, and it’s this little box and it’s got this head on it, like with a camera. And you talk to it and it, like, lights up, it points at you has a screen, and you can ask it questions and you can have what I was hoping it would do is while I go, I’m gone, like running errands or I’m on the road or whatever I can call in, seen my mom and check on her and talk back and forth.” (Dennis)	

#### RQ3: Concerns and Challenges

Older adults specified several concerns and challenges that influenced their use of technology and AI. These barriers impacted their adaptation to new devices and their ongoing worries about the technology already associated with their routines.

##### Commitment to Learning

Most older adults articulated that the time and energy to learn new technology was not a worthwhile investment. Patricia remarked, “I’m sort of like this with technology, I use what I have to use...Now, if I wasn’t retired, I’d be embracing it a thousand percent because it would be necessary. But yeah, I don’t need to be.” Patricia differentiated the time period of retirement as 1 in which people no longer needed to keep up with innovations. Instead, she reiterated that her current use was at a level that she was comfortable continuing at. Other older adults shared this view, like Sandra who limited her activity on social media, stating:

I use Instagram and Facebook, but I just review it. It turns out every time I try to say anything on it, I don’t say what I mean. I said happy anniversary to somebody once, but I sent them a picture of a pizza. If that gives you an idea why I don’t type on Instagram.

Sandra focused on completing online tasks that matched her skill level with the tool. She wanted to review the content to stay connected on interpersonal updates but following miscommunication with the addition of a meme, she limited her participation. Older adults like Patricia and Sandra were motivated to use technology with their current abilities but did not prioritize allocating time and effort to using new technologies or improving their capacity with existing offerings.

##### Lack of Privacy

Another concern that older adults expressed was how AI technologies could threaten their personal privacy. Given the growth of technology throughout their lifetime and the rapid change of the AI landscape in the last few years, many stated concerns about insufficient regulation regarding these digital tools. Anthony conveyed this uneasiness, saying:

Part of the problem, I think, is people don’t realize how much of their rights they’re signing away when they just click through or the acknowledge or whatever...>With digital data, some of its okay, I’m not worried about the fact that I’ve got, you know, this Google doc or that Google Sheet or whatever...[B]ut what do you think about what’s going on in your household? And you know, and think about some things, some artificial intelligence in this instance that’s listening and maybe sending information all the time about everything.

Anthony compared content that had a minimal need for privacy with parts of his life he considered deserving of more extensive restrictions. Anthony was 1 of multiple older adults who questioned if bringing technology into their home was worth the risk of sharing information and communication about their household with technology companies. Often older adults were not only worried about their own privacy but also articulated that these considerations were likely not considered by other, less technologically knowledgeable older adults.

##### Dependence on Technology

Older adults also discussed their fear of technology replacing their performance of daily tasks which would ultimately lead to cognitive decline. This concern was shared among older adults who were concerned that technology was an assistant for laziness and that relying on these tools reflected a negative moral character. Exemplifying this, Gwen explained:

Probably as I get older, I might could use some more support. But I also feel that I’m a big believer if you don’t use what you have, you lose it, so if I don’t use the knowledge I have, or the memory I have, or some of that, then it just goes off because you’re not helping that muscle to keep developing.

Gwen offers an analogy of exercising her brain with tasks that keep her mind active. This analogy leaves technology as a cheat tool that limits this exercise from taking place. Older adults integrated the language of exercise while expressing their worries that technology would replace the routines they had in place to support their brain health.

Overall, older adults expressed their concerns with learning new technologies, the lack of privacy related to the implementation of these technologies, and the impact that these devices would have on their health. These concerns informed how older adults were oriented toward technology and AI regarding their willingness to use these digital devices.

#### RQ4: Attitudes Toward Technology

##### Overview

Older adults reported having diverse attitudes toward technology and AI. These orientations influence the degree to which older adults were willing to engage with technology and the attributes they described technology with. The orientations ranged from *adapters* who were willing and able to use a range of technology to support their life to *resisters,* who were adults who articulated their aversion to integrating technology into their daily lives. The adaptor and resister orientations represented the opposing positions and incorporating aspects of both orientations, some older adults described a *wary* disposition.

##### Adapters

Adapters to technology and AI encompassed participants who were most willing and excited to merge digital devices into their everyday routines. Many older adults embraced the positive attributes and the assistance that technology brought to their lives. For instance, Christine remarked:

When you stop and think about what my parents had to do to get through a day, I’m almost embarrassed that my life is so easy. It’s like Thursday’s the day to turn on the robotic vacuum cleaner. And I think, okay, I dont even have to remember to put on the dishwasher. You know, you’re just embarrassed that people had to physically do this work and take up all day. And it’s so easy for us...Our last two homes, we did have thermostats we could do with our phone and, you know, all of that. [W]e use Ring. You gotta love Ring. You don’t really want to talk to strangers at the door, so we use a lot of different tools.

Christine’s remark highlights the extent to which she has integrated technology into her life, including the use of automation and smart home devices. Many older adults emphasized how the use of technology decreased the burden of once time-consuming tasks which was a reward for integrating new digital tools. Technologies were so integral to some older adults’ lives that 1 participant reflected, “Sometimes I think she’s [Alexa] my best friend” (Deborah). Deborah was 1 of multiple older adults using generative AI systems like Alexa for interpersonal connection. This connection fostered regular connection such that a friendship connection grew. Adapters of technology integrated technology and AI into their daily routines with such enthusiasm that the benefits of such adaptation were clear to older adults.

##### Wary

Some older adults were hesitant to use technology or were unsure of the advantages that the integration of digital devices would bring to their lives. Most older adults with this orientation were using new(er) technologies but were less confident about their usage of digital devices. For instance, Raymond compared his technology knowledge with his wife’s, stating:

I think she’s [wife] less afraid of making a mistake. She’s just not as afraid as me. I’m always a little wary of it, I go by the rule that if it’s not broken, don’t fix it, you know. But that doesn’t get you any further ahead. But it keeps you at that level rather than falling back, but I don’t move ahead.

Gregory emphasized the caution with which approaches new technologies. Gregory concluded that he felt more comfortable continuing to use technology in the same way as before instead of learning or incorporating new interfaces. This was a sentiment shared by other older adults like Clarence, who shared,

I tend to view it [artificial intelligence] as a negative because you know, if you click on a couple articles, then all of a sudden you’re being fed all these articles and it can support a view that maybe you were just interested in, but then all of a sudden it’s giving you, you know stuff that supports maybe someone else’s point of view, it wasn’t really yours. But the more you’re inundated with this information the more you tend to believe it.

Clarence pointed out how AI could facilitate access to information consistent with his worldview yet also overwhelm him with content inconsistent with his perspective. For Clarence, AI was central to targeted algorithm manipulation that impacted what he saw online. This belief left Clarence, and many others, feeling skeptical about AI and the application of the tool in his life. When introduced to other benefits of using AI, Clarence pondered,

Oh, like reminders for medications and workout schedules and meal suggestions. I didn’t realize that that stuff was out there, but I pretty much eat salad and chicken and that’s, you know, fruits and vegetables, healthy diet.

This participant was among a group of older adults who were interested in learning about previously unknown aspects of technology and AI that might support their daily routines, while also acknowledging some concerns about adopting new technologies. These older adults were hesitant to adopt new technologies but were not opposed to learning about new features or watching important others use digital devices.

##### Resistant

The final orientation toward technology and AI was older adults who were resistant to the adoption and integration of digital devices in their lives. Participants with this orientation reported that they avoided learning new technologies and often wished that they could return to a time in their life before these advancements. Speaking on this, Donna articulated:

“I basically don’t like it...I also think it takes away from personal interaction, which is harmful to humanity...I don’t like it. I kind of rather go back to, you know, people farming and taking care of themselves. I’m a homesteader, so I really don’t like technology very much. I know we use it every day and it is helpful in that way. But I basically don’t like it...I kind of basically ignore it and don’t use it if I don’t have to. If I have to, then I have to. But if I don’t need to, then I don’t.

Donna articulated a sense of nostalgia and skepticism toward technology, positioning these advancements as contrary to traditional ways of life. Ultimately, she expressed an aversion to technology and AI as she concluded that she would only use these devices when necessary. This orientation was shared by other older adults who were unwilling or unable to incorporate technology and AI. This sentiment was central to 1 participant’s perspective because this resistant orientation had led to him losing his job. Raymond recounted:

And when I turned 62, they said, we really can’t keep you. You can get Social Security if you want, or you can get a job, but we can’t keep you. And part of the criticism I heard about me was I wasn’t technologically savvy enough and I didn't have any interest.

Raymond reflected on the termination of his position related to his unwillingness to integrate new technologies. Raymond recounted that he remained focused on completing his job with the tools he learned when training as an accountant and was indifferent to studying new adaptations of software that he was able to apply to his work. Resisters encompassed older adults who were satisfied with their current knowledge and who were unwilling to consider future integrations regardless of the possibilities that technology and AI offered to their lives.

## Discussion

### Principal Results and Comparison With Previous Work

The first research question aimed at understanding the current use of technology and AI by older adults. All older adults were using some form of technology, and most were using a combination of smartphones, smartwatches, and computers in their daily routines. Older adults specified that their current use of technology aided them in social interaction, entertainment, and health management, and assisted them in productivity tasks. This is consistent with findings from past research, which has documented similar uses of technology among older adults. For example, the use of digital devices for maintaining social connections, such as through social media and video calls [33]. Similarly, the use of technology for health management, including fitness trackers and health apps has been widely reported [34].

The second research question was focused on what technology older adults would be interested in learning and integrating into their lives. These results were crowdsourced from older adults to ensure that any future AI technology tools could incorporate aspects of technology that were interesting and helpful. Participants articulated that helpful technology would provide schedule reminders, help with laborious chores, and reduce their caregiving load by monitoring loved ones. These findings are consistent with previous research, which has highlighted older adults’ interest in practical, supportive technologies. For example, previous studies have shown that older adults value technologies that enhance their independence, such as devices that provide medication reminders or manage daily schedules [35,36]. Interestingly, although we aimed to examine the use of technology to benefit older adults’ health and well-being, many participants expressed interest in technology to monitor others’ health and lighten the load of informal caregiving. This reflects both the diversity of health and independence in older age and the need to develop technologies that can be applied by older adults both for their own well-being and to monitor the health of others [37,38]. To better meet the needs of older adults, developers should prioritize implementing user-centered designs to tailor to older adults’ preferences [39]. For instance, participatory design approaches involve older adults early in the design process as “co-designers” [40]. This approach ensures their insights and feedback are incorporated, leading to technological solutions that accurately reflect their preferences and needs.

Related to the third research question, our results revealed that the notable barriers to technology and AI use were related to older adults’ lack of commitment to learning new technology, privacy concerns, and fear of becoming too dependent on technology. Older adults have expressed hesitancy to learn new technology, mentioning that the time and energy required do not seem worthwhile. Such hesitancy is often linked to their perception of high learning effort expectancy [41]. This suggests that some older adults view mastering a new system as overly demanding. Thus, to address the perception that technology is too demanding for older adults, it is essential to enhance the perceived ease of use. Providing clear, step-by-step guides on how to use these technologies can play a crucial role in making them more accessible and less intimidating for this demographic. Another barrier found in our study, privacy concerns, has been brought up in previous studies as a significant barrier to technology adoption [8,41,42]. Especially for voice-based systems, older adults reported that they are not comfortable with devices that may have access to and store their personal conversations [8,38,41]. Finally, some of our participants expressed fear that reliance on technology may replace their performance of daily tasks, leading to cognitive decline. Similar concerns were reported in previous research where older adults reported skepticism about using technology potentially deterring their memory [43]. For developers, it is crucial to recognize the cognitive and physical changes that come with aging to design inclusive and user-friendly systems [44]. Previous work suggests that aging-driven designs may effectively address challenges such as isolation and physical changes for older adults [45]. This approach focuses on tailoring technological solutions to the specific problems faced by older adult users.

Finally, our findings of the fourth research question reveal that not all older adults are resistant to technology and AI. Rather, the spectrum of technology adoption among older adults can be broad, encompassing adapters, weary, and resistant individuals. Previous studies have found that these variations could be influenced by individual factors such as age, education, and other sociodemographic factors. For instance, familiarity with technology was not a significant barrier for relatively young older adults (aged 65-75 years) who are already active users of smartphones, whereas older adults aged >75 years were more likely to face barriers toward adopting technology [8]. In addition, evidence shows that higher education and living with a spouse or partner were positively associated with increased use of information communication technology [46]. Therefore, to encourage greater adoption of new technologies, such as AI-based communication systems, it is crucial to consider individual factors and tailor solutions to meet the diverse circumstances of older adult users.

### Limitations

Limitations for all studies should be considered. First, this study did not distinguish between age groups of older adults including young-old (65-74 years), old-old (75-84 years), and oldest-old (over 85 years old) [38]. The age of older adults likely impacts their orientation and willingness to accept new technologies. For instance, some participants in the sample worked in offices before the widespread integration of computers. Learning technology as a requirement of one’s employment might impact the way that older adults view technological advancement compared with others who only voluntarily interacted with technologies throughout their adulthood. Future researchers might differentiate between age groups to examine how the orientations outlined are applicable across sections of older adults. Another limitation of this study pertains to self-selection bias. Recruiting older adults through a community research pool may attract participants who are more technologically adept and familiar with technology, potentially underrepresenting those who face technological barriers or hold negative attitudes toward technology [47]. To address this limitation, future research should adopt recruitment strategies, such as using multiple recruitment channels and partnering with organizations (eg, local senior centers), to be more inclusive toward individuals with limited access to or resistance to technology [36,48].

In addition, older adults routinely experience stereotypes and stigma related to agism [46,49]. This ageism might limit an individual’s willingness to adopt a learning mindset with new technology given the vulnerability of labeling a knowledge deficient. Examining how stereotypes serve as barriers for older adults integrating technology and seeking support would inform researchers on how to decrease the spread of this harmful communication and combat the negative implications of the messages through public health campaigns targeted at empowering technology use for members of this community.

### Conclusions

Despite the increasing interest and potential benefits of using AI technology for older adult populations, significant challenges persist in the current technology landscape. The current study aimed to deepen our understanding of how older adults perceive technology and AI while also identifying challenges and opportunities for improvement. Our findings suggest a critical need for developing more personalized and aging-friendly systems that can be successfully integrated into older adults’ daily lives. Moving forward, it is essential to keep the dialogue open between developers, older adults, their families, and their care team to ensure that the design of AI technologies is inclusive and supportive to older adults.

## Abbreviations

AI: artificial intelligence
BRFSS: Behavioral Risk Factor Surveillance System

## Data Availability

The data generated and analyzed during this study are available from the corresponding author on reasonable request.

## References

[1] (2020). WHO methods and data sources for life tables 1990-2019. World Health Organization.

[2] (2023). Mental health of older adults. World Health Organization.

[3] Teo RH, Cheng WH, Cheng LJ, Lau Y, Lau ST (2023). Global prevalence of social isolation among community-dwelling older adults: a systematic review and meta-analysis. Arch Gerontol Geriatr.

[4] Ruggiano N, Brown EL, Roberts L, Framil Suarez CV, Luo Y, Hao Z, Hristidis V (2021). Chatbots to support people with dementia and their caregivers: systematic review of functions and quality. J Med Internet Res.

[5] Sapci AH, Sapci HA (2019). Innovative assisted living tools, remote monitoring technologies, artificial intelligence-driven solutions, and robotic systems for aging societies: systematic review. JMIR Aging.

[6] Rodríguez-Martínez A, Amezcua-Aguilar T, Cortés-Moreno J, Jiménez-Delgado JJ (2023). Qualitative analysis of conversational chatbots to alleviate loneliness in older adults as a strategy for emotional health. Healthcare (Basel).

[7] Chou YH, Lin C, Lee SH, Chang Chien YW, Cheng LC (2023). Potential mobile health applications for improving the mental health of the elderly: a systematic review. Clin Interv Aging.

[8] Gudala M, Ross MET, Mogalla S, Lyons M, Ramaswamy P, Roberts K (2022). Benefits of, barriers to, and needs for an artificial intelligence-powered medication information voice chatbot for older adults: interview study with geriatrics experts. JMIR Aging.

[9] Hülür G, Macdonald B (2020). Rethinking social relationships in old age: digitalization and the social lives of older adults. Am Psychol.

[10] Olson KE, O'Brien MA, Rogers WA, Charness N (2011). Diffusion of technology: frequency of use for younger and older adults. Ageing Int.

[11] Latikka R, Rubio-Hernández R, Lohan ES, Rantala J, Nieto Fernández F, Laitinen A, Oksanen A (2021). Older adults' loneliness, social isolation, and physical information and communication technology in the era of ambient assisted living: a systematic literature review. J Med Internet Res.

[12] Marziali RA, Franceschetti C, Dinculescu A, Nistorescu A, Kristály DM, Moșoi AA, Broekx R, Marin M, Vizitiu C, Moraru S, Rossi L, Di Rosa M (2024). Reducing loneliness and social isolation of older adults through voice assistants: literature review and bibliometric analysis. J Med Internet Res.

[13] GPT-3.5-Turbo-0125 Model. OpenAI.

[14] Kocaballi AB, Sezgin E, Clark L, Carroll JM, Huang Y, Huh-Yoo J, Kim J, Kocielnik R, Lee YC, Mamykina L, Mitchell EG, Moore RJ, Murali P, Mynatt ED, Park SY, Pasta A, Richards D, Silva LM, Smriti D, Spillane B, Zhang Z, Zubatiy T (2022). Design and evaluation challenges of conversational agents in health care and well-being: selective review study. J Med Internet Res.

[15] SeniorTalk - Chatbot for Elderly People.

[16] Broadbent E, Loveys K, Ilan G, Chen G, Chilukuri MM, Boardman SG, Doraiswamy PM, Skuler D (2024). ElliQ, an AI-driven social robot to alleviate loneliness: progress and lessons learned. JAR Life.

[17] Emmer De Albuquerque Green C (2024). Defining responsible use of AI chatbots in social care for older adults. Nat Aging.

[18] Elavsky S, Knapova L, Janiš K, Cimler R, Kuhnova J, Cernicky T (2024). Multiple perspectives on the adoption of SMART technologies for improving care of older people: mixed methods study. J Med Internet Res.

[19] Peek STM, Wouters EJ, Luijkx KG, Vrijhoef HJ (2016). What it takes to successfully implement technology for aging in place: focus groups with stakeholders. J Med Internet Res.

[20] National Academies of Sciences (2020). Engineering, and Medicine. Social Isolation and Loneliness in Older Adults: Opportunities for the Health Care System.

[21] Chawla K, Kunonga TP, Stow D, Barker R, Craig D, Hanratty B (2021). Prevalence of loneliness amongst older people in high-income countries: a systematic review and meta-analysis. PLoS One.

[22] Park C, Majeed A, Gill H, Tamura J, Ho RC, Mansur RB, Nasri F, Lee Y, Rosenblat JD, Wong E, McIntyre RS (2020). The effect of loneliness on distinct health outcomes: a comprehensive review and meta-analysis. Psychiatry Res.

[23] Ong AD, Uchino BN, Wethington E (2016). Loneliness and health in older adults: a mini-review and synthesis. Gerontology.

[24] Dahlberg L, McKee KJ, Frank A, Naseer M (2022). A systematic review of longitudinal risk factors for loneliness in older adults. Aging Ment Health.

[25] Jensen V, Clarke V (2006). Using thematic analysis in psychology. Qualitative Research in Psychology.

[26] Kidder LH (1981). Research Methods in Social Relations.

[27] Lincoln YS, Guba EG, Pilotta JJ (1985). Naturalistic Inquiry.

[28] Corbin J, Strauss A (2008). Basics of Qualitative Research: Techniques and Procedures for Developing Grounded Theory.

[29] Centers for Disease Control and Prevention (2020). A data users' guide to the disability questions included in the behavioral risk factor surveillance system.

[30] Hughes ME, Waite LJ, Hawkley LC, Cacioppo JT (2004). A short scale for measuring loneliness in large surveys: results from two population-based studies. Res Aging.

[31] Diener E, Emmons RA, Larsen RJ, Griffin S (1985). The satisfaction with life scale. J Pers Assess.

[32] Yoon J, Lee M, Ahn JS, Oh D, Shin S, Chang YJ, Cho J (2022). Development and validation of digital health technology literacy assessment questionnaire. J Med Syst.

[33] Kaur A, Chen W (2023). Exploring AI literacy among older adults. Stud Health Technol Inform.

[34] Chopik WJ (2016). The benefits of social technology use among older adults are mediated by reduced loneliness. Cyberpsychol Behav Soc Netw.

[35] Heart T, Kalderon E (2013). Older adults: are they ready to adopt health-related ICT?. Int J Med Inform.

[36] Mitzner TL, Boron JB, Fausset CB, Adams AE, Charness N, Czaja SJ, Dijkstra K, Fisk AD, Rogers WA, Sharit J (2010). Older adults talk technology: technology usage and attitudes. Comput Human Behav.

[37] Vaportzis E, Clausen MG, Gow AJ (2017). Older adults perceptions of technology and barriers to interacting with tablet computers: a focus group study. Front Psychol.

[38] Dodge HH, Du Y, Saxton JA, Ganguli M (2006). Cognitive domains and trajectories of functional independence in nondemented elderly persons. J Gerontol A Biol Sci Med Sci.

[39] Dopp AR, Parisi KE, Munson SA, Lyon AR (2019). A glossary of user-centered design strategies for implementation experts. Transl Behav Med.

[40] Lindsay S, Jackson D, Schofield G, Olivier P (2012). Engaging older people using participatory design.

[41] Min SH, Topaz M, Lee C, Schnall R (2023). Understanding changes in mental health symptoms from young-old to old-old adults by sex using multiple-group latent transition analysis. Geroscience.

[42] Barnard Y, Bradley MD, Hodgson F, Lloyd AD (2013). Learning to use new technologies by older adults: perceived difficulties, experimentation behaviour and usability. Computers in Human Behavior.

[43] Bonilla K, Martin-Hammond A Older adults' perceptions of intelligent voice assistant privacy, transparency, and online privacy guidelines.

[44] Vines J, Pritchard G, Wright P, Olivier P, Brittain K (2015). An age-old problem examining the discourses of ageing in HCI and strategies for future research. ACM Trans Comput-Hum Interact.

[45] Carroll JM, Convertino G, Farooq U, Rosson MB (2011). The firekeepers: aging considered as a resource. Univ Access Inf Soc.

[46] Wang S, Bolling K, Mao W, Reichstadt J, Jeste D, Kim HC, Nebeker C (2019). Technology to support aging in place: older adults' perspectives. Healthcare (Basel).

[47] Poli A, Kelfve S, Berg K, Motel-Klingebiel A (2021). Old-age diversity is underrepresented in digital health research: findings from the evaluation of a mobile phone system for post-operative progress monitoring in Sweden. Ageing and Society.

[48] Peek ST, Luijkx KG, Rijnaard MD, Nieboer ME, van der Voort CS, Aarts S, van Hoof J, Vrijhoef HJ, Wouters EJ (2016). Older adults' reasons for using technology while aging in place. Gerontology.

[49] Vroman KG, Arthanat S, Lysack C (2015). 'Who over 65 is online?' Older adults’ dispositions toward information communication technology. Computers in Human Behavior.

